# Clinical value of the adjustable suspension technique in laparoscopic pyeloplasty

**DOI:** 10.3389/fsurg.2026.1743710

**Published:** 2026-04-13

**Authors:** Long-Yao Xu, Ling-Ling Chen, Wen-Hua Huang, Chao-Ming Zhou, Xu Cui, Liu Chen

**Affiliations:** 1 Department of Urology, Fujian Children’s Hospital (Fujian Branch of Shanghai Children’s Medical Center), College of Clinical Medicine for Obstetrics & Gynecology and Pediatrics, Fujian Medical University, Fuzhou, China; 2 Department of Oncology Gynecology, Clinical Oncology School of Fujian Medical University, Fujian Cancer Hospital, Fuzhou, China

**Keywords:** adjustable suspension, isolated UPJO in children, laparoscopic pyeloplasty, minimally invasive fascia closure device, single-line pelvis suspension

## Abstract

**Objective:**

The aim of our study was to explore the efficacy and safety of adjustable suspension in laparoscopic pyeloplasty (ASLP) and single-line pelvis suspension in laparoscopic pyeloplasty (SLPSLP) for isolated ureteropelvic junction obstruction (UPJO) in children.

**Methods:**

We retrospectively reviewed the clinical data of all the children with isolated UPJO who underwent laparoscopic pyeloplasty (LP) at Fujian Children's Hospital between July 2019 and July 2023. We collected data from the medical records of all the children and analysed their clinical characteristics as well as their operative and follow-up data.

**Results:**

One hundred thirteen children with isolated UPJO who had complete clinical data and underwent LP at our centre were included in this study. Fifty-two children underwent ASLP and 61 children underwent SLPSLP successfully at our centre. There was no statistically significant difference in patient age, sex, body weight, proportion of patients with a history of UTI or pain, follow-up time or side of the UPJO between the ASLP group and the SLPSLP group (*P* > 0.05). However, there were significant differences in the operation duration, blood loss volume, time to DJ stent extraction, time to abdominal drainage tube extraction and length of hospitalisation between the ASLP group and the SLPSLP group (*P* < 0.05). The APD and PT before surgery were significantly different from those 6 and 12 months after surgery in both the ASLP and SLPSLP groups (*p* < 0.05). The anastomotic leakage rate was significantly different between the two groups, as no patients in the ASLP group and 6 patients in the SLPSLP group experienced anastomotic leakage (*P* = 0.030). Anastomotic stenosis occurred in 1 patient in the ASLP group and 4 patients in the SLPSLP group, with no significant difference between the two groups (*P* = 0.372).

**Conclusion:**

Compared with SLPSLP, ASLP is advantageous in that it is minimally invasive, has a shorter operation time, and causes less bleeding. Thus, compared with conventional LP, adjustable suspension in laparoscopic pyeloplasty involving a minimally invasive fascia closure device for paediatric isolated UPJO is safe and effective.

## 1 Introduction

Ureteropelvic junction obstruction (UPJO) is one of the most common causes of hydronephrosis in children, and the most commonly used and effective treatment at present is Anderson Hynes dismembered pyeloplasty, which is considered the “gold standard” for the treatment of UPJO in children (1). Owing to the development of minimally invasive technology, laparoscopic pyeloplasty (LP) has become the preferred surgical modality because of its advantages such as less trauma, faster recovery, and less pain, and its success rate is similar to that of open pyeloplasty (OP) (2). However, LP in children is difficult to learn, not to mention time-consuming, owing to intracorporeal suturing in the narrow space of the intraperitoneal cavity (3). Since its initial introduction in 1996 by Tan et al., the single-line suspension technique (4) has been widely used and optimized to improve intraoperative exposure, thus reducing the difficulty of both the laparoscopic procedure and anastomosis (5, 6).

However, the single-line suspension technique is known for limited exposure of the surgical field, and the use of additional suspension wires is associated with injury to both the renal pelvis and ureteral sheath. In addition, multilayer suspension affects the blood supply at the anastomosis site, which is not conducive to anastomotic healing after surgery and increases the possibility of postoperative scar stenosis (7). Similar to the study by Luo YH (7), the retrospective analysis by Cui X (8) revealed that in the 66 infants with UPJO included, 3–0 absorbable sutures placed in the upper abdomen successfully lifted the upper edge of the antetheca of the pelvis, leading to adequate exposure of the renal pelvis; however, the direction and depth of the suspension could not be adjusted arbitrarily. As a result, both the laparoscopic procedure and anastomosis remained difficult. Therefore, the data of 113 children with isolated UPJO admitted to our hospital between July 2019 and July 2023 were retrospectively analysed in this study. In this study, a minimally invasive fascia closure device was used for adjustable suspension in LP, allowing easy manipulation of the renal pelvis and ureter in all directions, and LP with single-line pelvis suspension was also performed. The aim of our study was to explore the efficacy and safety of laparoscopic pyeloplasty with adjustable suspension for isolated ureteropelvic junction obstruction in children.

## 2 Materials and methods

### 2.1 Study population

This study was approved by the ethics committee of Fujian Children's Hospital (Approval No. 2025ETKLRK01003). All the patients' guardians signed an informed consent form before the operation. We identified patients with isolated UPJO who underwent single-line pelvis suspension in laparoscopic pyeloplasty (SLPSLP) or adjustable suspension in laparoscopic pyeloplasty (ASLP) between July 2019 and July 2023. A minimally invasive fascia closure device produced by Xiamen Surgaid Medical Equipment Co., Ltd., of China (Patent No. ZL 2013 20013865.2) was used for adjustable suspension of the renal pelvis or ureter. Finally, we retrospectively analysed the clinical data of 113 patients who were treated for isolated UPJO in our hospital between July 2019 and July 2023, including preoperative, intraoperative, postoperative and follow-up data.

The inclusion criteria were as follows: (1) patients who presented with isolated UPJO; (2) had indications for surgery, such as clinical symptoms related to hydronephrosis, including pain or urinary tract infection; an initial evaluation revealing a decline in renal function on the affected side to <40% with a renal tubular half-life >20 min; exacerbation of hydronephrosis or persistent hydronephrosis accompanied by thinning of the renal parenchyma, as observed on follow-up ultrasound; or an APD of the renal pelvis >3.0 cm or ≥2.0 cm with calyceal dilatation; (3) underwent LP; and (4) had complete and accessible medical records and follow-up data.

The exclusion criteria were as follows: (1) patients who had bilateral UPJO, complicated with stones or other congenital deformities, such as horseshoe kidney, duplex kidney and vesicoureteral reflux (VUR); (2) had a history of posterior abdominal surgery and a high risk of developing difficult-to-separate abdominal adhesions; (3) had severe renal dysfunction or an isolated kidney; (4) had serious cardiopulmonary diseases and coagulopathy; or (5) refused to sign the consent form for surgery or refused to comply with the follow-up schedule.

### 2.2 Surgical methods

This was a simple retrospective controlled review of case records at our centre. Two operations have been performed in our department for many years, and the technology has been improved. Patients were divided into two groups on the basis of the procedure performed, one of which was ASLP, which is minimally invasive, and the other was SLPSLP, which is the traditional technique. All patients were operated on by two chief physicians of equal seniority. Notably, the selection of surgical technique was surgeon preference–based: one surgeon tended to perform ASLP, while the other tended to perform conventional SLPSLP.

#### 2.2.1 Adjustable suspension in laparoscopic pyeloplasty (ASLP)

**Figure 1:**
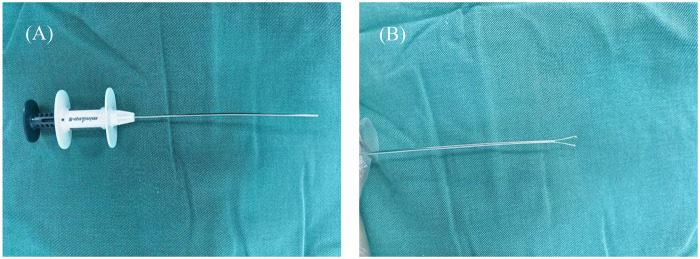
A minimally invasive fascia closure device **(A)** the minimally invasive fascia closure device apparatus; **(B)** the magnifed image of the two-hooks in the core distal end.

**Figure 2:**
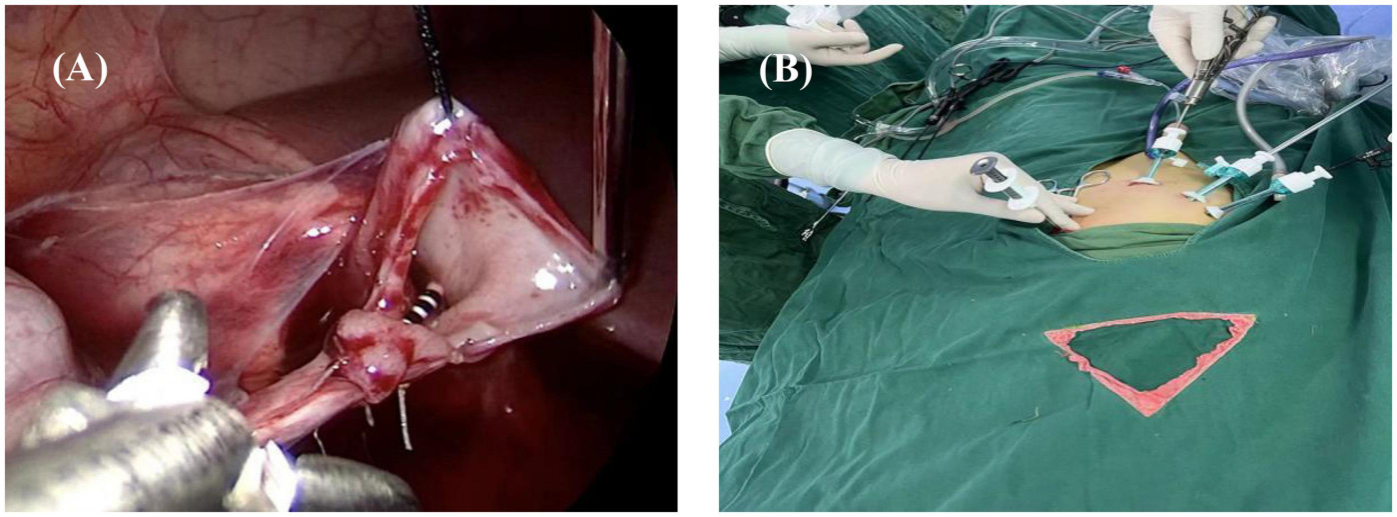
Images of adjustable suspension in laparoscopic pyeloplasty apparatus. **(A)** The minimally invasive fascia closure device clamp was placed in the upper abdomen to assist accurately pyeloureteral anastomosis by pulling the pyelostomy margin. **(B)** The minimally invasive fascia closure device and trocha position diagram.

**Figure 3:**
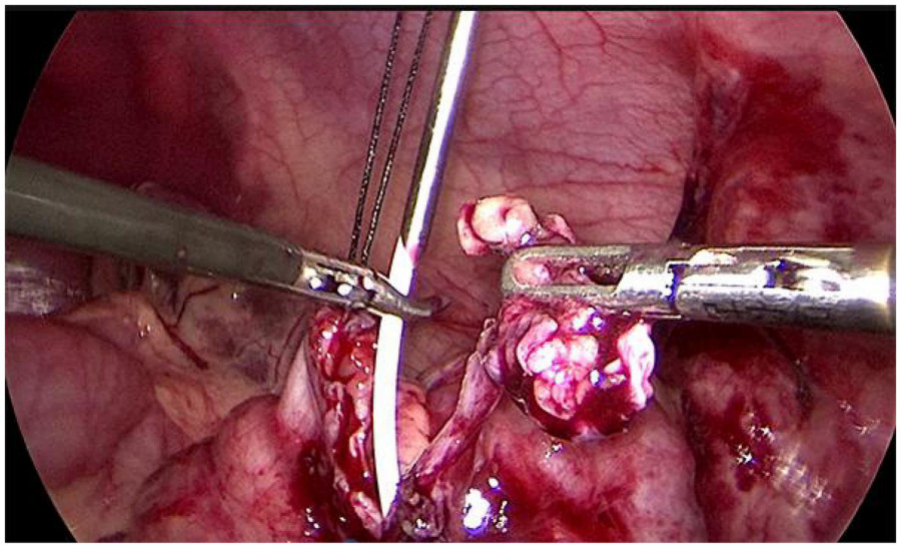
Images of the guide wire and double J tube were inserted through the outer sheath of the pneumoperitoneum needle,which is the place of the puncture point of the original minimally invasive fascia closure.

ASLP involves pelvis or ureter suspension with a minimally invasive fascia closure device (Figures 1A,B), which was produced by Xiamen Surgaid Medical Equipment Co., Ltd. of China (Patent No. ZL 2013 20013865.2), for exposing the surgical area. During the laparoscopic and anastomosis procedures, the clamp of the fascia closure device can grasp the renal pelvis or ureter in all directions, providing sufficient traction and ensuring better exposure without affecting the blood supply to the anastomosis (Figure 2A). The operations were performed through three ports, one with a 5 mm trocar and two with 3 mm trocars. (Following the diamond rule, Figure 2B). The renal pelvis was suspended at the corresponding body surface point approximately 2 cm above the UPJ with a 3–0 puncture needle. The minimally invasive fascia closure device was placed between the first suspension line and the anastomosis to lift the upper edge of the antetheca at the ureter approximately 2 cm from the UPJ for complete ureteropelvic suspension. The stenosed segment of the ureter was removed, and then we made a longitudinal incision measuring 1.5 cm in the ureter, and a “tongue-shaped” flap was formed. After the lowest point of the renal pelvis and the lowest point of the ureteral longitudinal incision were identified, the minimally invasive fascia closure device was used as auxiliary forceps to assist anastomosis of the anterior and posterior walls of the ureteropelvic junction. Notably, the outer sheath of the pneumoperitoneum needle was placed at the original puncture point selected for minimally invasive fascia closure, and both the guide wire and double J tube were inserted to assist in the rapid placement of the ureteral stent (Figure 3). The abdominal cavity was flushed with saline, and an 8-F silicone abdominal drainage tube with many side openings was placed via the trocar channel into the hypogastrium.

#### 2.2.2 Single-line pelvis suspension in laparoscopic pyeloplasty (SLPSLP)

For single-line pelvis suspension in laparoscopic pyeloplasty (SLPSLP), a 3–0 absorbable suture is placed in the upper abdomen to lift the upper edge of the antetheca of the pelvis. The operations were performed through three ports—one with a 5 mm trocar and two with 3 mm trocars. Only the renal pelvis was suspended at the corresponding body surface point approximately 2 cm above the UPJ with a 3–0 puncture needle. The stenosed segment was subsequently identified and resected. An approximately 1.5 cm incision was made longitudinally, proximal to distal from the outer side of the ureter from proximal to distal using scissors, and a “tongue-shaped” flap was formed. After the lowest point of the renal pelvis and the end of the ureteral longitudinal incision was identified, the flap and both the anterior and posterior walls of the renal pelvis were sutured with 5–0 absorbable sutures. The guide wire and DJ tube were inserted through the outer sheath of the pneumoperitoneum needle, which was inserted between the first suspension line and the anastomosis. The renal pelvis was then sequentially sutured with 5–0 absorbable sutures. The abdominal cavity was flushed with saline, and an 8-F silicone abdominal drainage tube with many side openings was placed via the trocar channel into the hypogastrium (8).

### 2.3 Follow-up schedule

Preoperative and intraoperative clinical data were collected from operative reports and medical records. The postoperative clinical data included the change in both the APD and PT as well as the incidence of anastomotic leakage or stenosis. All the patients were followed up for more than 12 months. The change in anteroposterior pelvic diameter (APD) was calculated as the value of preoperative APD minus postoperative APD, and the change in parenchymal thickness (PT) was defined as postoperative PT minus preoperative PT. The drainage tube was routinely removed when the daily drainage volume was less than 10–20 mL, with no evidence of fluid leakage or infection. Urinary system and abdominal ultrasonography was performed to exclude significant hydronephrosis and obvious ascites before removal. The final timing of drain removal was determined based on comprehensive clinical manifestations, laboratory examinations, and imaging findings. The per urethral catheter was routinely removed on the second day after abdominal drain removal. The Double-J stent was removed when postoperative ultrasound showed no significant hydronephrosis or urinary extravasation, and the patients had no evidence of urinary tract infection.

### 2.4 Statistical analysis

Statistical analysis was conducted using SPSS 19.0 (SPSS Inc., Chicago, IL, USA). The chi-square test was used to compare qualitative variables; other comparisons were made using appropriate statistical tests. Student's *t* test was performed to compare continuous variables. Variance analysis was performed to compare the mean values between the two groups. A *P* value < 0.05 was considered to indicate statistical significance.

## 3 Results

Fifteen patients without complete clinical data and 8 patients with incomplete follow-up data were excluded, leaving a total of 113 isolated UPJO patients with complete clinical data who underwent LP at our centre. Fifty-two patients underwent ASLP and 61 patients underwent SLPSLP successfully at our centre.

There were was no statistically significant difference in patient age, sex, body weight, proportion of patients with a history of UTI or pain, follow-up time or side of the UPJO between the **ASLP** group and the **SLPSLP** group (*P* = 0.995, 0.791, 0.921, 0.550, 0.645 and 0.411, respectively). There was no significant difference between the two groups in terms of the PT or APD before surgery (*p* > 0.05). The characteristics of the patients are described in Table 1.

**Table 1:** Clinical characteristics of the ASLP and SLPSLP groups before operation.

Characteristics	ASLP	SLPSLP	*t* (*χ*^2^) value	*P* value	Effect size (95% CI)
Age (months) (Mean ± SD, range)	29.42 ± 27.26, (1.03–85.00)	29.45 ± 27.04, (0.73–85.00)	T = 0.006	0.995	MD = 0.033, 95% CI (−10.119, 10.185)
Gender, *n* (%)			χ^2^ = 0.119	0.791	OR = 0.830, 95% CI (0.288, 2.392)
Boys	44 (84.6%)	53 (86.9%)			
Girls	8 (15.4%)	8 (13.1%)			
Side, *n* (%)			χ^2^ = 0.938	0.411	OR = 1.489, 95% CI (0.670, 3.480)
Right	18 (65.4%)	16 (26.2%)			
Left	34 (34.6%)	45 (73.8%)			
Weight (kg) (Mean ± SD, range)	12.38 ± 5.78, (4.00–23.00)	12.49 ± 5.43, (4.50–23.00)	T = 0.100	0.921	MD = 0.106, 95% CI (−1.987, 2.198)
History of UTI or pain, *n* (%)			χ^2^ = 0.403	0.550	OR = 0.772, 95% CI (0.347, 1.717)
Yes	15 (28.8%)	21 (34.4%)			
NO	37 (71.2%)	40 (65.6%)			
APD before surgery (cm) (Mean ± SD, range)	3.34 ± 0.79, (2.00–6.00)	3.57 ± 1.07, (2.00–7.50)	T = 1.300	0.196	MD = 0.233, 95% CI (−0.122, 0.589)
PT before surgery (cm) (Mean ± SD, range)	0.24 ± 0.09, (0.11–0.67)	0.26 ± 0.12, (0.10–0.75)	T = 0.958	0.340	MD = 0.020, 95% CI (−0.021, 0.061)
Fllow up time (months) (Mean ± SD, range)	16.07 ± 5.07, (12.00–36.77)	15.63 ± 4.94, (12.00–29.30)	T = −0.462	0.645	MD = −0.436, 95% CI (−2.307, 1.435)

Single-line pelvis suspension in Laparoscopic Pyeloplasty (SLPSLP) and adjustable suspension in Laparoscopic Pyeloplasty (ASLP). Parenchymal thickness (PT) and anteroposterior diameter (APD); Categorical variables are represented as number (%) and continuous variables as mean ± standard deviation (range).

The blood loss volume, operation duration, length of the stenosed ureteral segment, diameter of the stenosed ureteral segment, length of hospitalisation, time to DJ stent removal, time to abdominal drainage tube removal, hospitalisation costs, change in APD and PT in the two groups are shown in Table 2. There were statistically significant differences in the operation duration, blood loss volume, time to DJ tube extraction, abdominal drainage tube extraction time and length of hospitalisation between the ASLP group and the SLPSLP group (*P* = 0.019, 0.014, 0.011, 0.002 and 0.012, respectively), whereas there was no significant difference in the length of the stenosed ureteral segment, diameter of the stenosed ureteral segment, the value of APD change at 3 months after surgery, the value of PT change at 3 months after surgery or hospitalisation costs between the two groups (*p* > 0.05). In contrast, the APD and PT before surgery were significantly different from those 6 and 12 months after surgery in both the ASLP and SLPSLP groups (*p* < 0.05).

**Table 2:** The intraoperative and postoperative conditions were compared between the ASLP and SLPSLP groups.

Characteristics	ASLP	SLPSLP	*t* (χ^2^) value	*P* value	Effect size (95% CI)
Length of ureter stenosis (cm); Mean ± SD (Range)	1.38 ± 0.40, (0.50–3.50)	1.41 ± 0.29, (1.00–2.50)	T = 0.336	0.737	MD = 0.022, 95% CI (−0.107, 0.151)
Diameter of ureteral stenosis (cm); Mean ± SD (Range)	0.126 ± 0.045, (0.10–0.30)	0.129 ± 0.050, (0.05–0.30)	T = 0.284	0.777	MD = 0.003, 95% CI (−0.015, 0.020)
Operation duration (minutes), Mean ± SD (Range)	117.71 ± 17.28, (82–190)	126.36 ± 20.82, (85–190)	T = 2.377	0.019	MD = 8.649, 95% CI (1.440, 15.858)
Bleeding volume, (mL), Mean ± SD (Range)	5.63 ± 2.76, (2.00–16.00)	7.36 ± 4.31, (2.00–25.00)	T = 2.484	0.014	MD = 1.726, 95% CI (0.349, 3.103)
DJ stent extraction time(day) Mean ± SD (Range)	48.48 ± 11.14, (30.00–70.00)	53.87 ± 10.91, (30.00–76.00)	T = 2.592	0.011	MD = 5.388, 95% CI (1.269, 9.507)
Abdominal drainage tube extraction time(day) Mean ± SD (Range)	3.58 ± 1.05, (3.00–7.00)	4.89 ± 2.75, (3.00–21.00)	T = 3.237	0.002	MD = 1.308, 95% CI (0.508, 2.109)
Length of hospitalization (day)	7.04 ± 2.43, (5.00–15.00)	8.59 ± 3.77, (5.00–30.0)	T = 2.548	0.012	MD = 1.548, 95% CI (0.344, 2.753)
Hospitalization costs (yuan), Mean ± SD (Range)	30,016.40 ± 5,393.53, (23,122–52,105)	29,979.94 ± 5,469.81, (22,788–55,688)	T = −0.044	0.965	MD = −45.455, 95% CI (−2,078.150, −1,987.240)
Postoperative APD change at 3 months (mm) Mean ± SD (Range)	18.40 ± 10.22, (−11.00–52.00)	19.0 ± 12.64, (−6.00–67.00)	T = 0.308	0.759	MD = 0.673, 95% CI (−3,661, 5.008)
Postoperative PT change at 3months (mm) Mean ± SD (Range)	1.16 ± 1.12, (−2.40–3.00)	1.13 ± 0.89, (−1.60–3.30)	T = −0.141	0.888	MD = −0.027, 95% CI (−0.401, 0.347)
Postoperative APD change at 6 months (mm) Mean ± SD (Range)	23.91 ± 8.89, (−4.00–53.50)	19.84 ± 11.78, (1.00–60.00)	T = −2.043	0.043	MD = −4.067, 95% CI (−8.013, −0.122)
Postoperative PT change at 6 months (mm) Mean ± SD (Range)	1.80 ± 1.17, (−2.20–3.50)	1.38 ± 0.96, (−1.50–3.40)	T = −2.113	0.037	MD = −0.423, 95% CI (−0.820, −0.026)
Postoperative APD change at 12 months (mm) Mean ± SD (Range)	26.66 ± 10.00, (−12.00–53.00)	22.20 ± 12.77, (−3.00–67.00)	T = −2.044	0.043	MD = −4.467, 95% CI (−8.798, −0.136)
Postoperative PT change at 12 months (mm) Mean ± SD (Range)	2.43 ± 1.20, (−1.80–3.90)	1.93 ± 1.06, (−1.20–4.70)	T = −2.334	0.021	MD = −0.495, 95% CI (−0.916, −0.075)

Single-line pelvis suspension in Laparoscopic Pyeloplasty (SLPSLP) and adjustable suspension in Laparoscopic Pyeloplasty (ASLP). Parenchymal thickness (PT) and anteroposterior diameter (APD); Categorical variables are represented as number (%) and continuous variables as mean ± standard deviation (range).

The postoperative complications in the ASLP and SLPSLP groups are shown in Table 3. In this study, the difference in the anastomotic rate was significantly different between the two groups, as no patients in the ASLP group and 6 patients in the SLPSLP group experienced anastomotic leakage (*P* = 0.030). There was no significant difference in the anastomotic stenosis, postoperative intestinal obstruction, or postoperative urinary tract infection rate between the two groups (*P* = 0.372, 1.000 and 0.623, respectively). Two patients in the ASLP group and only 1 patient in the SLPSLP group developed calculi postoperatively, and the difference in the rate of postoperative renal calculi development was not significant between the two groups (*P* = 0.594).

**Table 3:** Postoperative complications in the ASLP and SLPSLP groups.

Complications, *n*(%)	ASLP	SLPSLP	Statistical Test	*P* value	Effect Size (Phi)	95% CI (OR)
Leakage of the anastomosis, *n* (%)	0 (0%)	6 (9.8%)	Fisher's exact test	0.030	0.3	(1.12, ∞)
Anastomotic stenosis, *n* (%)	1 (1.9%)	4 (6.6%)	Fisher's exact test	0.372	0.11	(0.41, 31.20)
Postoperative intestinal obstruction, *n* (%)	0 (0%)	1 (1.6%)	Fisher's exact test	1.000	0.09	(0.00, 32.80)
Postoperative urinary tract infection, *n* (%)	1 (1.9%)	3 (4.9%)	Fisher's exact test	0.623	0.08	(0.27, 25.50)
Postoperative Calculin (%)	2 (3.8%)	1 (1.6%)	Fisher's exact test	0.594	0.07	(0.04, 4.40)

Single-line pelvis suspension in Laparoscopic Pyeloplasty (SLPSLP) and adjustable suspension in Laparoscopic Pyeloplasty (ASLP). Parenchymal thickness (PT) and anteroposterior diameter (APD); Categorical variables are represented as number (%) and continuous variables as mean ± standard deviation (range).

## 4 Discussion

UPJO is a prevalent cause of hydronephrosis in children, contributing to 85%–90% of cases (9). Since it was first reported in 1949 (10), conventional Anderson‒Hynes pyeloplasty has served as the gold standard for managing UPJO. Despite its success rate exceeding 90%, OP requires a large incision and is known for postoperative pain, a long recovery, and poor aesthetic outcomes (3). However, on the contrary, LP is widely and successfully performed in various countries, becoming one of the preferred methods for managing UPJO as the incision required for the operation is easily concealed, meaning minimal scarring, and the procedure is minimally invasive, meaning a shorter operation time, minimal bleeding, mild pain, and rapid recovery (11). Nevertheless, LP can be difficult and time-consuming for junior physicians to master, especially in children, as intracorporeal suturing can be challenging because of the small intraperitoneal cavity in children (12).

Considering that anastomoses are difficult to create in narrow spaces, some physicians have opted to create more puncture holes to improve visibility of the surgical field; however, because this approach increases the number of surgical incisions, postoperative healing is often delayed and and the aesthetic outcome is poor (13). In several cases, physicians have used fewer trocars to minimize scarring and therefore achieve a good cosmetic outcome. Moreover, relevant research has led to the replacement of traditional single-line suspension (4) and single-line pelvis suspension (14) with double-line renal suspension (6) and the latest layered multi-point suspension (15), both of which are considerable improvements. In 1996, Tan et al. (4) proposed the application of single-line suspension in laparoscopic dismembered pyeloplasty. Although this method is associated with better exposure of the operative field, it is also known for its association with injury to the renal pelvis and ureter. Mandelia A (14) and Yu XF (15) reported that increasing the number of hierarchical suspension lines improves visibility of the operative field to some extent, which is conducive to laparoscopic cutting and anastomosis; however, the direction and depth of the suspension lines cannot be adjusted at any time, such as with auxiliary forceps, which are not better for anastomosis and cutting. Conversely, excessive suspension affects the blood supply of the renal pelvis and ureter. Cui X (8) conducted a retrospective analysis of the clinical data of 66 infants with UPJO from our hospital and reported that single-line pelvis suspension in laparoscopic pyeloplasty is safe and feasible for the treatment of UPJO in children. Similar to a previous study by Chen J (16), our preliminary research revealed that the single-line suspension method is simple to perform and does not require repeated puncture or suturing of the renal pelvis and ureter, thus reducing the risk of damage to the renal pelvis and ureter. However, only a few suspension lines were used, thus exposure of the ureteropelvic region was poor, and the two ends of the ureteropelvic anastomosis was malpositioned. Therefore, auxiliary exposure, tailoring and anastomosis are limited. Moreover, our latest study differed in that a mini gripper was sourced from the fascia closure device, which was produced by Xiamen Surgaid Medical Equipment Co., Ltd., of China (Patent No. ZL 2013 20013865.2) and can be adjusted in all directions to manipulate the renal pelvis or ureter, such as with auxiliary forceps, to improve the feasibility of the procedure, shorten the operative and suturing times, and improve overall comfort.

To our knowledge, the renal APD and PT measured by B-ultrasound are mainly used for evaluating the recovery of the diseased kidney after surgical treatment for hydronephrosis and are significantly correlated with the glomerular filtration rate (GFR) of the ipsilateral kidney, which can effectively reflect renal function (17).Menon reported that patients recovered renal function within 3 months after the operation (18). FERNáNDEZ IBIETA M (19) reported that kidney function can return to normal within approximately 5 months after surgery. However, Rivas JG (20) reported that the 1-year follow-up diuretic renography revealed recovery of renal function in some patients. Therefore, all patients were followed up for more than 1 year in this study. Notably, The APD and PT before surgery were not significantly different from 3 months after surgery in both the ASLP and SLPSLP groups. The reason may have been due to the oedema stage in the first 3–6 months after surgery (21). If the case of hydronephrosis is relatively stable and there are no obvious clinical symptoms, regular follow-up can be continued. Conversely, if the condition worsens, a double J tube or a nephrostomy tube may be reinserted.

Compared with those in the single-line suspension group, the operation time was shorter, the incidence of postoperative anastomosis was lower, and the APD and PT were more significantly improved at 6 and 12 months after the operation in the adjustable suspension group, confirming that the adjustable suspension technique was safe and effective. Although the use of a fascia closure device increased the total cost of the procedure, the difference in the total cost of each procedure was not significant due to the shorter length of hospitalisation and the shorter time to abdominal drainage tube extraction, as well as the reduced incidence of postoperative complications in the ASLP group. Moreover, the adjustable suspension technique is an easy to learn, minimally invasive approach associated with easier anastomosis creation in the narrow intraperitoneal cavity of a child, lower risk of intraoperative bleeding and fewer incisions, meaning fewer scars and a better aesthetic outcome.

In July 2019, we introduced a novel, modified technique called the adjustable suspension technique for treating paediatric UPJO. The technique is minimally invasive, involves the use of a fascia closure device, and has been successfully performed on more than 52 children with isolated UPJO over the last 4 years, with few complications. Studies have revealed that anastomotic stenosis is one of the main complications after pyeloplasty (22). Anastomotic stenosis is closely related to the technique used for intraoperative anastomosis. High-density sutures can cause postoperative ischaemia, oedema, and poor healing at the anastomotic site, and low-density sutures can cause urine leakage, stimulate the surrounding tissue to induce an inflammatory response, and cause scar tissue hyperplasia. Finally, excessive tension at the anastomosis, excessive pyelotomy and excessive free tissue around the ureter can also cause anastomotic stenosis (23). In this study, only 1 case of anastomotic stenosis occurred in the adjustable suspension group. Unfortunately, the difference in the incidence of anastomotic stenosis was not statistically significant between the two groups, possibly because of the short follow-up time and small sample size.

Moreover, studies have revealed that the main causes of postoperative anastomotic leakage include excessive clamping for anastomosis creation, stent obstruction, and improper placement of the drainage tube. Protecting the blood flow to the anastomosis, adjusting the position of the drainage tube and resolving any stent obstruction are crucial for improving symptoms (24). The adjustable suspension group had a significantly lower incidence of anastomotic leakage than the single-line suspension group did. A minimally invasive fascial closure device was used instead of a clamp for anastomosis creation and as a second auxiliary clamp for accurate cutting, greatly reducing the incidences of anastomotic stenosis and anastomotic leakage.

On the basis of our experience, we noted the following advantages of using this technique:

The Single-line pelvis suspension was feasible for most surgeons performing LP, and the learning curve for the procedure is quite short; however, the direction and depth of suspension cannot be adjusted arbitrarily. Although the double-line suspension technique could increase the accuracy of anastomosis creation, the tension of the suspension line should be carefully controlled. Without adequate tension, the suspension line will be loose and therefore not conducive to anastomosis creation. In contrast, excessive tension might cause the tissue at the suspension line to tear, leading to postoperative scar tissue hyperplasia and possibly anastomotic stenosis. Our latest study revealed that adding a minimally invasive fascial closure device, such as a second auxiliary forceps, for single-line pelvis suspension could allow arbitrary adjustment of the suspension line without increasing the risk of pyeloureteral injury or number of trocars needed and therefore incisional scars. The tip of the needle is sharp, and the body of the needle is round in shape, allowing easy passage through tissues without major abrasions or cuts. The clamp can act as auxiliary forceps, helping to pull the renal pelvis or ureter, achieving arbitrary adjustments of the direction and depth of suspension. This method provides stable fixation and good exposure of the renal pelvis and ureter, making the opening of the ureter easy to find and cutting along the suspension line more precise.

The adjustable suspension technique involves less tension between the pelvis and ureter, allowing the creation of a tension-free anastomosis and reducing the probability of anastomotic leakage and anastomotic stenosis. Moreover, using a minimally invasive fascia closure device as auxiliary forceps provides stable fixation of the free ureter and allows the surgeon to use his/her left hand for anastomosis creation, thus shortening the overall cutting and anastomosis creation times as well as reducing the difficulty of the procedure. The posterior wall is not easily anastomosed because of the blocking of the ureteral tongue flap, if the anterior wall has been anastomosed. The device can grasp the ends for end-to-end pyeloureteral anastomosis without damaging the blood supply.

Similarly, Cao HL's (25) study revealed that excessive clamping increased the risk of anastomotic leakage. In our study, mini double-hook forceps, which are primarily used for fascia closure, were passed through a needle-sized incision for clipping and clamping the pyeloureteral anastomosis. In addition, compared with ordinary auxiliary forceps, mini double-hook forceps have a lower clamping force, cause less damage to anastomotic tissue, and are more conducive to anastomotic healing.

In this study, the puncture point for minimally invasive fascia closure had to be carefully selected. The puncture point had to suitable to accommodate a pneumoperitoneum needle for double J tube placement and auxiliary forceps for cutting and anastomosing the renal pelvis and ureter. Therefore, the puncture point selected in this study was between the first suspension line and the anastomosis, which reduced both the number of punctures and the number of scars. It is well known that there is no absolute uniform standard for the timing of double-J stent removal after pyeloplasty for pediatric UPJO. The decision is primarily individualized based on the surgical approach, intraoperative conditions, patient age, and postoperative recovery. The core principle is to ensure anastomotic healing while minimizing stent indwelling time to reduce the risk of complications. In our study, while ASLP was associated with a statistically significant reduction in the time to double-J stent removal relative to SLPSLP, implying a potential advantage, the substantive clinical significance of this difference remains unclear and warrants further research.

Our study also has a few limitations. First, this was a single-centre study, thus multicentre studies are needed for further assessment of the effectiveness and complications of this technique. Second, this was a retrospective review, and the sample size was small. Third, the difference in the incidence of anastomotic stenosis was not significantly different between the two groups, as 4 patients in the SLPSLP group and only 1 patient in the ASLP group experienced anastomotic stenosis, possibly because of the short follow-up time. Thus, as the short follow-up time is another limitation of the study, studies with a longer follow-up are needed for further analyses of the possible causes of anastomotic stenosis. A notable limitation of this study is the lack of objective renal functional assessment via diuretic renography, which was unavailable for pediatric patients in the early study period due to institutional equipment constraints. Thus, the evaluation of renal recovery was restricted to ultrasound-derived APD and parenchymal thickness, and all inferences regarding renal function recovery were correspondingly conservative. Although some perioperative outcomes (e.g., reduction in operative time, lower blood loss and shorter DJ stent extraction time) showed statistically significant differences, the absolute magnitudes were relatively small, suggesting modest rather than clinically decisive advantages. Another important limitation is that the two surgical techniques were predominantly performed by two different surgeons, which may introduce surgeon-related confounding factors. Although baseline characteristics were well balanced between groups, surgeon-specific experience, technical habits, and performance may independently influence surgical outcomes, particularly anastomotic leakage. Therefore, the observed difference in anastomotic leakage should be interpreted as hypothesis-generating rather than definitive evidence of superiority or inferiority of either technique. Conclusions regarding clinical efficacy should be drawn cautiously, and further randomized controlled studies with standardized surgical performance and surgeon stratification are warranted to confirm these findings.

## 5 Conclusion

Our study revealed that, compared with SLPSLP, ASLP was advantageous in terms of minimal invasiveness, a shorter operation time, minimal bleeding. In conclusion, compared with conventional LP, adjustable suspension LP involving a minimally invasive device typically used for fascia closure is safe and effective for paediatric isolated UPJO.

## Data Availability

The dataset in this study comprises clinical medical records, which are restricted by patient privacy protection regulations and the ethical review guidelines of the affiliated institution. Thus, the dataset cannot be publicly shared or disclosed. Any requests for data access should be submitted to the institutional ethical review board and must comply with relevant legal and ethical requirements. The data that support the findings of this study are available from the corresponding author upon reasonable request at fjsetyycl@163.com.
